# A biomimetic and pH-sensitive polymeric micelle as carrier for paclitaxel delivery

**DOI:** 10.1093/rb/rbx023

**Published:** 2017-08-16

**Authors:** Boxuan Ma, Weihua Zhuang, Gongyan Liu, Yunbing Wang

**Affiliations:** 1 National Engineering Research Center for Biomaterials, Sichuan University, Chengdu 610064, China and; 2 National Engineering Laboratory of Clean Technology of Leather Manufacture, Department of Biomass Chemistry, Sichuan University, Chengdu 610064, China

**Keywords:** pH sensitive, polymeric micelles, drug delivery, biomimetic

## Abstract

As nano-scale drug delivery systems, smart micelles that are sensitive to specific biological environment and allowed for target site-triggered drug release by reversible stabilization of micelle structure are attractive. In this work, a biocompatible and pH-sensitive copolymer is synthesized through bridging poly (2-methacryloyloxyethyl phosphorylcholine) (PMPC) block and poly (_D, L_-lactide) (PLA) block by a benzoyl imine linkage (*Blink*). Biomimetic micelles with excellent biocompatibility based on such PLA-*Blink*-PMPC copolymer are prepared as carriers for paclitaxel (PTX) delivery. Due to the rapid breakage of the benzoyl imine linkage under acidic condition, the micelle structure is disrupted with accelerated PTX release. Such pH-sensitive triggered drug release behavior in synchronization with acidic conditions at tumor site is helpful for improving the utilization of drug and facilitating antitumor efficacy. These micelles can be used as promising drug delivery systems due to their biocompatible and smart properties.

## Introduction

Nano-polymeric drug carriers, such as micelles, have been extensively applied in cancer therapy for their significantly reduced side effects and improved therapeutic efficacy. Particularly, a proper diameter of less than 200 nm can promote drug-loaded micelles to be passively accumulated into tumor tissue benefiting from the enhanced permeability and retention (EPR) effect, which indicates a potential for higher utilization ratio of drug and great convenience in chemotherapy [1–3]. However, as a drug delivery system, these micelles must be endowed with several featured properties during the treatment such as escaping from rapid renal clearance by reticuloendothelial system (RES), keeping longevity in blood circulation, rapidly releasing the cargo in target site and degrading into biosafety ingredients [4–6].

In order to avoid recognizing by RES, biocompatible Poly (ethylene glycol) (PEG) based surface has been widely developed due to its compact associations with water molecules via hydrogen bond, which forms a hydrating layer and gives ‘stealth’ property to the micelles [7–9]. Besides conventional PEG, polymers containing zwitterionic phosphorylcholine have got increasingly attentions due to their superior biocompatibility, strong biostability and remarkable resistant to protein adsorption in recent years [10, 11]. For instance, poly (2-methacryloyloxyethyl phosphorylcholine) (PMPC) has been introduced into several nano-polymeric drug carriers as hydrophilic blocks and exhibited great performance [12–15]. Different from PEG, PMPC copolymer achieves high hydration by ionic solvation, which plays an important role in its non-fouling property [16, 17]. Therefore, PMPC can be used as a promising block candidate for fabricating biomimetic drug delivery system [18–20].

Ideally, drug-loaded micelles should possess not only great biocompatibility and stability in blood stream, but also intelligent property for controlled releasing the payload at target sites [21–25]. Therefore, smart micelles designed with responsive features have been applied for target site-triggered releasing payload drugs in synchronization with specific biological environments, e.g. acidic conditions in tumor tissues (pH∼6.8) or in late endosome/lysosome in cells (pH < 5.5) [4, 26–32]. Especially, several chemical bonds, such as hydrazones which are known to be stable at neutral pH but susceptible to be hydrolyzed at acidic pH, have been used to covalently conjugate drugs into micelles for pH-triggered release [5]. For example, Kataoka and co-workers have constructed a smart prodrug micelle with a hydrazone connecting the anticancer drug and the amphiphilic copolymer, which would be stable at physiological pH but susceptible to hydrolytic cleavage at tumor microenvironment [33]. Moreover, several pH-sensitive bonds including acetal [34–38], imine [39], orthoester [40, 41] have also been introduced as block linkage for designing smart micelles on account of their rapid bond breakage at tumor acidic condition. Among these chemical bonds, benzoyl imine linkage has exhibited stronger stability compared with traditional imine linkage, which attributes to the π-π conjugation provided by benzene ring but still maintain the pH-sensitivity inheriting from its imine moity [42, 43].

**Scheme 1:**
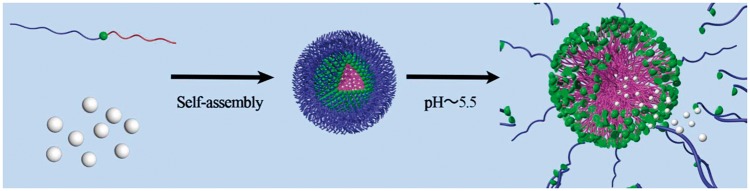
Illustration of the PTX-loaded micelle with pH sensitive property as a drug carrier and its disintegration under acid environment

In this work, amphiphilic diblock copolymer designed by bridging poly (_D, L_-lactide) (PLA) and poly (2-methacryloyloxyethyl phosphorylcholine) (PMPC) via benzoyl imine linkage (*Blink*) was synthesized for biomimetic and pH-sensitive micelles. Furthermore, the hydrophobic PLA block was terminated by cinnamyl alcohol which was supposed to improve drug loading content, owing to the π-π staking between the benzene ring of cinnamyl alcohol and paclitaxel (PTX) [4]. In addition, the PTX-loaded micelles showed great stability in normal physiological environment but quickly disintegrated under acidic environment due to the breakage of the benzoyl imine linkage, resulting in triggered drug release (Scheme 1).

## Experiments section

### Materials

2-Methacryloyloxyethyl phosphorylcholine was purchased from Nanjing Natural Science and Technology Institute and used without further purification. _D, L-_Lactide and stannous octoate were purchased from Sigma-Aldrich. Paclitaxel (semisynthetic), cinnamyl alcohol, 4-carboxy benzaldehyde, 2-Bromoisobutyryl bromide, dicyclohexylcarbodiimide (DCC), dimethylaminopyridine (DMAP), N-Boc-ethylenediamine and 2,2'-dipyridyl (bpy) were purchased from Chengdu Best Reagent Co., LTD (Chengdu, China) and used as received. Cuprous bromide (CuBr) from Chengdu Best Reagent Co., LTD (Chengdu, China) was purified by washing with acetic acid and ethanol. Methylbenzene and dimethyl formamide (DMF) (from Chengdu KeLong Chemical Reagent Company (Chengdu, China)) were purified by distilled. All other reagents and solvents were purchased from Chengdu KeLong Chemical Reagent Company (Chengdu, China) and used without further purification.

### Synthesis of benzaldehyde conjugated poly (_D, L_-lactide) (CPLA-CHO)

First, _D, L_-lactide (3 g, 21 mmol), cinnamyl alcohol (73.3 mg, 54.6 mmol), stannous octoate (85 mg, 0.21 mmol) and dry methylbenzene (1.5 ml) were added in a dry tube. After three times of freeze-pump-thaw procedure, the tube was sealed under vacuum and the mixture was heated to 130 °C and stirred for 3 h. The crude product was dissolved in dichloromethane, concentrated and then precipitated into excess cold ethyl ether and dried in vacuum at room temperature overnight. ^1 ^H NMR (400 MHz, CDCl_3_, δ): 7.3 ppm (5 H, C_6_H_5_CH-), 6.65 ppm (H, C_6_H_5_CH-), 6.2-6.3 ppm (H, C_6_H_5_CHCH-), 4.79 ppm (2 H, -CHCH_2_-), 5.2 ppm (nH, -OCOCH(CH_3_)-), 1.5 ppm (3nH, -OCOCH(CH_3_)-).

Then, poly (_D, L_-lactide) (2 g, 0.4 mmol), 4-carboxybenzaldehyde (90 mg, 0.6 mmol), DCC (136.2 mg, 0.66 mmol) and DMAP (100 mg, 0.82 mmol) were dissolved in dry DMF (40 ml) and added in a dry round-bottom flask. After stirred at room temperature for 72 h, the solution was concentrated and the product was collected by precipitated into excess cold ethyl ether and dried in vacuum at room temperature overnight. ^1 ^H NMR (400 MHz, CDCl_3_, δ): 7.3 ppm (5 H, C_6_H_5_CH-), 6.7 ppm (H, C_6_H_5_CH-), 6.2-6.3 ppm (H, C_6_H_5_CHCH-), 4.79 ppm (2 H, -CHCH_2_-), 5.2 ppm (nH, -OCOCH(CH_3_)-), 1.5 ppm (3nH, (-OCOCH(CH_3_)-), 7.9 ppm (4 H, -C_6_H_4_CHO), 10.1 ppm (H, -C_6_H_4_CHO).

### Synthesis of amino-terminated poly (2-methacryloyloxyethyl phosphorylcholine) (PMPC-NH_2_)

The initiator N-Boc-(2-aminoethyl)-2-bromo-2-methylpropanamide was synthesized according to our previous report [44]. Then the hydrophilic polymer PMPC was synthesized by ATRP method. Typically, initiator (0.1545 g, 0.5 mmol) and MPC (4 g, 13.5 mmol) were dissolved in a dry flask with 40 ml methanol (MeOH). After three times of freeze-pump-thaw procedure, CuBr (72 mg, 0.5 mmol) and 2, 2'-dipyridyl (156.2 mg, 1.0 mmol) were added into the flask under the protection of N_2_. The reaction was performed at 40 °C for 48 h with N_2_ protection. The catalyst was removed by passing through a neutral aluminum oxide column. The solution was concentrated, precipitated into excess cold ethyl ether and dried in vacuum at room temperature overnight to get PMPC-N-Boc. ^1 ^H NMR (400 MHz, CD_3_OD, δ): 4.3 ppm (2nH, -COOCH_2_-), 4.25 ppm (2nH, -COOCH_2_CH_2_-), 4.1 ppm (2nH, -PO_4_CH_2_-), 3.75 ppm (2nH, -PO_4_CH_2_CH_2_-), 3.3 ppm (9nH, -CH_2_N(CH_3_)_3_), 1.4 ppm (9 H, C(CH_3_)_3 _O-).

The product mentioned above was deprotected by adding TFA (2 ml) in MeOH and stirred overnight. The final product was neutralized by aqueous sodium hydrogen carbonate, dialyzed against deionized water for 24 h (MWCO 3500) and freeze-dried. ^1 ^H NMR (400 MHz, CD_3_OD, δ): 4.3 ppm (2nH, -COOCH_2_-) 4.25 ppm (2nH, -COOCH_2_CH_2_-), 4.1 ppm (2nH, -PO_4_CH_2_-), 3.75 ppm (2nH, -PO_4_CH_2_CH_2_-), 3.3 ppm (9nH, -CH_2_N(CH_3_)_3_).

### Synthesis of *cinnamic-*poly (_D, L_-lactide)-*benzoyl imine link*-poly (2-methacryloyloxyethyl phosphorylcholine) (CPLA-*blink*-PMPC)

CPLA-CHO (0.5 g, 0.1 mmol) and PMPC-NH_2_ (0.8 g, 0.1 mmol) were added in a round-bottom flask with 20 ml dissolvent (MeOH/DMF, 1/1, v/v) and allowed to react for 48 h at room temperature. The product was purified by dialysis against deionized water for 24 h (MWCO 8000) and filtrated, followed by freeze-dried. ^1 ^H NMR (400 MHz, CD3OD/DMSO-d_6_ (1/1, v/v), δ): 5.2 ppm (nH, -OCOCH(CH_3_)-), 1.5 ppm (3nH, -OCOCH(CH_3_)-), 4.25 ppm (2nH, -COOCH_2_CH_2_-), 4.15 ppm (2nH, -PO_4_CH_2_-), 4.0 ppm (2nH, -PO_4_CH_2_CH_2_-), 3.25 ppm (9nH, -CH_2_N(CH_3_)_3_).

### Synthesis of *butyl*-PLA-*blink*-PMPC (PLA-*blink*-PMPC)

A control copolymer without an end group of benzene ring was synthesized by a similar approach. Especially, butyl alcohol was used instead of cinnamyl alcohol to initiate the polymerization of poly (_D, L_-lactide) and the final copolymer was endowed with a similar molecular weight with CPLA-*Blink*-PMPC.

### Preparation of PTX-loaded polymeric-micelles

Typically, CPLA-*Blink*-PMPC/PLA-*Blink*-PMPC (20 mg) and PTX (2 mg) were dissolved in 1 ml of methanol and acetonitrile (1/1, v/v) in a round-bottom flask. The solvent was evaporated under vacuum at 60 °C for 20 mins to form a film. Then PBS (20 ml) was added and the flask was shaken at 30 °C for 30 mins. The solution was subsequently extruded through a 0.45 μm syringe filter to get the PTX-loaded polymeric micelles (1 mg ml^−1^). The size of the PTX-loaded micelles was measured by Dynamic Light Scattering (DLS) and observed by Transmission Electron Microscopy (TEM) after stained with phosphotungstic acid. The influence of acid condition on the stability of PTX-loaded micelles was evaluated by incubating micelles solution at pH 5.5, and the particle size of the micelles was monitored by DLS at preselected time intervals. Drug loading content (DLC) and drug loading efficiency (DLE) were determined by high performance liquid chromatography (HPLC).

### *In vitro* drug release

The release behavior of PTX from PTX-loaded micelles were evaluated by dialysis. Typically, 2 ml solution of PTX-loaded micelles was added into a dialysis tube (MWCO 3500) with 100 ml PBS as release medium and then incubated at 37 °C in the dark with sustaining shake. At predetermined time intervals, 2 ml sample was taken out from the release medium and 2 ml fresh PBS was added. The sample was mixed with 2 ml acetonitrile and the released PTX was confirmed by HPLC.

### Cytotoxicity assay

The human cervical cancer (HeLa) cells and murine breast cancer (4T1) cells were cultured in DMEM and RPMI 1640 medium containing 10% (v/v) fetal bovine serum and 1% (w/v) penicillin-streptomycin at 37 °C in 5% CO_2_, 95% humidified atmosphere. The cytotoxicity of CPLA-*Blink*-PMPC copolymers, free PTX and PTX-loaded CPLA-*Blink*-PMPC micelles were evaluated by CCK-8 assays. HeLa and 4T1 cells were seeded into 96-well plates at a density of 8000 and 5000 per well with 200 μl medium. After 24 h, the medium was replaced with 200 μl fresh medium containing blank copolymers, free PTX and PTX-loaded micelles with different concentrations. The cells were incubated for 24 or 48 h and the medium was replaced by fresh medium containing 10% CCK-8 solution. After another 1.5 h of incubation, the absorbance was measured at a wavelength of 450 nm to evaluate the cytotoxicity.

### Cellular uptake

The cellular uptake assay was performed by confocal laser scanning microscopy (CLSM). Considering that PTX was invisible to the CLSM, fluorochrome Dil was loaded into the micelles to study the cellular uptake, following the earlier report of Wang et al. [45]. The 4T1 cells were seeded into glass bottom plates and cultured for 48 h. Then, the Dil-loaded micelles with the final Dil concentration of 0.5 μg ml^−1^ were added. After incubated for 2 h and 4 h, the medium was removed and the cells were washed with PBS for three times before observed by CLSM.

### Characterization

The chemical structures of the polymers were characterized by ^1 ^H NMR carried out on a 400 MHz NMR instrument (Bruker AMX-400). The polydispersity of CPLA-CHO and PMPC-NH_2_ were determined by gel permeation chromatography (GPC) (Agilent 1260). For CPLA-CHO, the measurements were performed by using DMF as the eluent at a flow rate of 1 ml min^−1^ at 40 °C and a series of narrow PS standards for the calibration. For PMPC-NH_2_, the eluent was H_2_O at a flow rate of 1 ml min^−1^ at 25 °C and a series of narrow PEG standards were used for calibration. The size and the morphology of micelles were characterized by a dynamic light scattering (DLS) and a Hitachi H-600 transmission electron microscope (TEM) with an accelerating voltage of 100 KV. High performance liquid chromatography (HPLC) (Agilent 1260) was used to determine the released drug. The measurements were performed by using acetonitrile/water (1/1, v/v) solvent as the eluent at a flow rate of 1 ml min^−1^ at 40 °C.

## Results and discussion

### Synthesis of CPLA-*blink*-PMPC copolymers

**Scheme 2:**
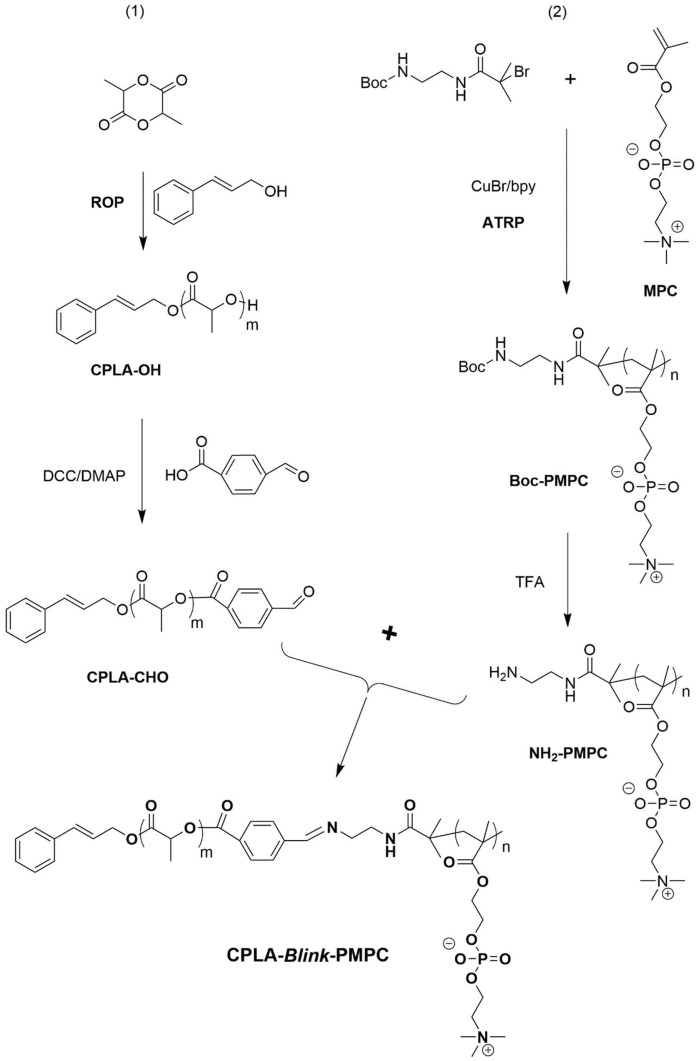
Synthetic route of CPLA-*blink*-PMPC copolymer

**Figure 1:**
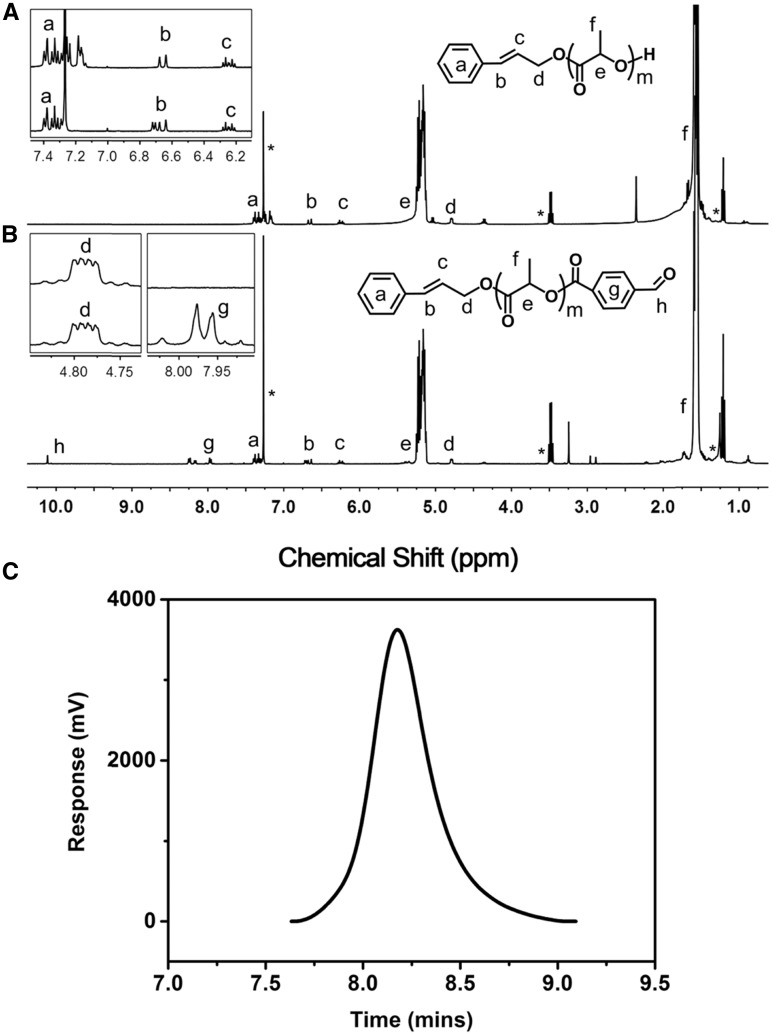
^1 ^H NMR Spectrum of CPLA-OH (**A**), CPLA-CHO (**B**) in CDCl_3_ and GPC spectra of CPLA-CHO in DMF (**C**)

The detailed synthetic route of CPLA-*Blink*-PMPC was shown in Scheme 2. First, hydrophobic CPLA-OH was synthesized through ring-opening polymerization (ROP) of _D, L_-lactide monomer by using cinnamyl alcohol as initiator. The ^1 ^H NMR spectrum of CPLA-OH was shown in Fig. 1A, in which the characteristic peaks of PLA (δ 5.2 ppm, -OCOCH(CH_3_)-; δ 1.5 ppm, -OCOCH(CH_3_)-) were found, while the characteristic peak of cinnamyl alcohol group at δ 4.79 ppm (-CHCH_2_-) could also be found. Further, the degree of polymerization (DP) of the CPLA block was calculated to be 69 based on the integral area ratio of peak d (δ 4.79 ppm, -CHCH_2_-) and peak f (δ 1.5 ppm, -OCOCH(CH_3_)-). Then 4-carboxy benzaldehyde was conjugated to CPLA-OH through esterification reaction with the terminal hydroxyl group of PLA. The characteristic peak g (δ 7.9 ppm, -C_6_H_4_CHO) and h (δ 10.1 ppm, -C_6_H_4_CHO) of the end group benzaldehyde on CPLA-CHO on Fig. 1B demonstrated the successful preparation of CPLA-CHO. In addition, molecular weight distribution (Mw/Mn) of CPLA-CHO block was determined to be 1.2 (Fig. 1C).

**Figure 2:**
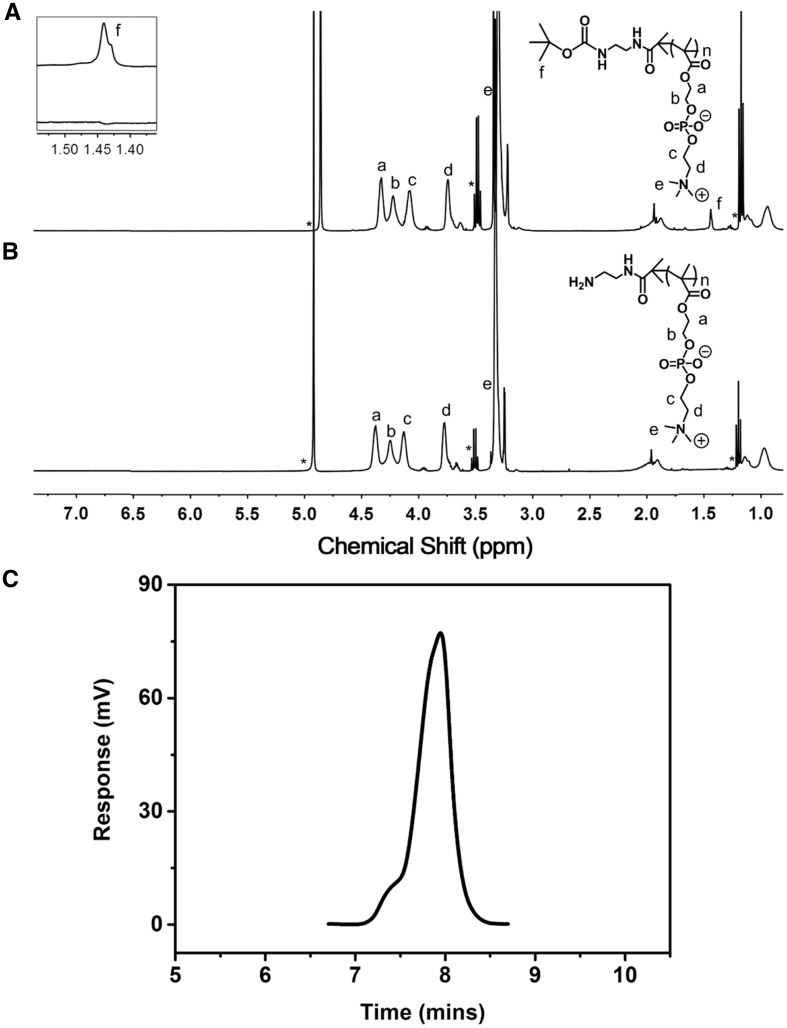
^1 ^H NMR Spectrum of PMPC-N-boc (**A**), PMPC-NH_2_ (**B**) in CD_3_OD and GPC spectra of PMPC-NH_2_ in H_2_O (**C**)

**Figure 3:**
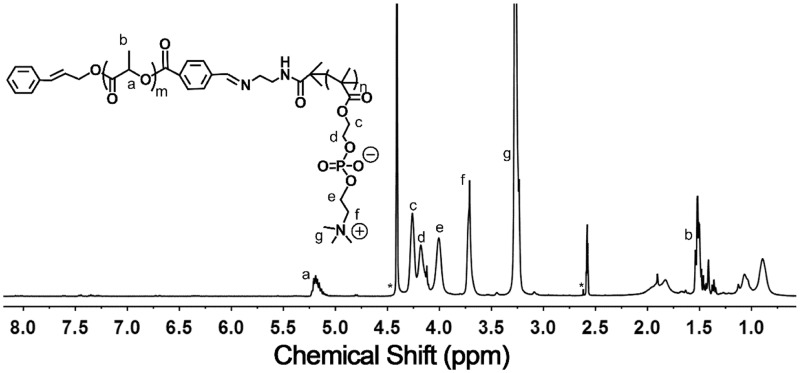
^1 ^H NMR Spectrum of CPLA-*blink*-PMPC copolymer in CD_3_OD/DMSO-d_6_ (1/1, v/v)

On the other hand, the characteristic peaks a-e in Fig. 2A (δ 4.3 ppm, -COOCH_2_-; δ 4.25 ppm, -COOCH_2_CH_2_-; δ 4.1 ppm, -PO_4_CH_2_-; δ 3.75 ppm, -PO_4_CH_2_CH_2_-; δ 3.3 ppm, -CH_2_N(CH_3_)_3_) were attributed to the hydrophilic polymer PMPC while the characteristic peak assigned to the initiator (δ 3.25 ppm, -CH_2_N(CH_3_)_3_) was found, suggesting the successful synthesis of PMPC-N-Boc. The DP of the PMPC block was calculated to be 27 based on the integral area ratio of peak f (δ 1.45 ppm, Boc) and peak d (δ 3.25 ppm, -CH_2_N(CH_3_)_3_). The Boc group was removed by trifluoroacetic acid and the disappearance of the peaks of the t-butyloxycarboryl at 1.45 ppm in ^1 ^H NMR results (Fig. 2B) confirmed the complete deprotection of the Boc group. Furthermore, the molecular weight distribution (Mw/Mn) of PMPC-NH_2_ was measured to be 1.15 (Fig. 2C) by GPC. Finally, CPLA-CHO and PMPC-NH_2_ were linked by a Schiff's based reaction. The ^1 ^H NMR result of benzoyl imine bridged copolymer was showed in Fig. 3 and the integral area ratio of peak b (δ 1.5 ppm, CPLA) and peak g (δ 3.25 ppm, PMPC) was 5:24, suggesting the successful synthesis of CPLA-*Blink*-PMPC. Therefore, the CPLA-*Blink*-PMPC synthesized in this work could be defined as CPLA_69_-*Blink*-PMPC_27_ with a molecular weight of about 13000 g mol^−1^.

The chemical structure of PLA-*Blink*-PMPC, synthesized as control copolymer, was characterized by ^1 ^H NMR and the molecular weight distribution was determined by GPC (Supplementary Figs. S1 and S2). The hydrophobic block PLA-CHO maintained a molecular weight of 5500 g mol^−1^ and a narrow distribution (Mw/Mn) of 1.2. Moreover, the final copolymer PLA-*Blink*-PMPC was endowed with a similar molecular weight of 13 300 g mol^−1^ with CPLA-*Blink*-PMPC, in order to study the encapsulation efficiency as compared with CPLA-*Blink*-PMPC.

### Structure and pH-response of PTX-loaded CPLA-*blink*-PMPC polymeric micelles

**Figure 4:**
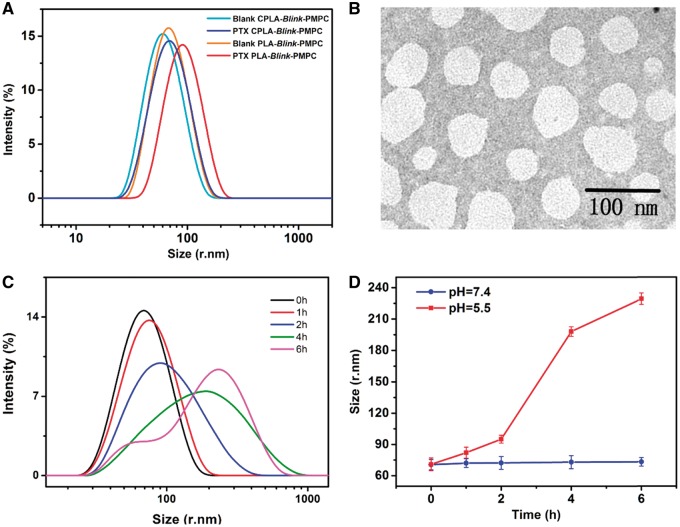
(**A**) The Particle size of blank and PTX-loaded CPLA-*blink*-PMPC and PLA-*blink*-PMPC micelles measured by DLS. (**B**) The size of PTX-loaded micelles detected by TEM. (**C**) Variation trend of particle size at pH 5.5 over time measured by DLS. (**D**) Size variation of PTX-loaded micelles at different pH over time measured by DLS

The particle size of CPLA-*Blink*-PMPC/PLA-*Blink*-PMPC micelles were measured by DLS while the drug loading content (DLC) and the drug loading efficiency (DLE) were determined by HPLC. The results were summarized in Table 1 and Fig. 4A. While the blank CPLA-*Blink*-PMPC and PLA-*Blink*-PMPC micelles had a similar size, CPLA-*Blink*-PMPC micelles exhibited smaller particle size (144 nm) than PLA-*Blink*-PMPC micelles (192 nm) when loaded PTX with the same drug/polymer weight ratios of 10%. Moreover, the PTX-loaded CPLA-*Blink*-PMPC micelles possessed higher DLC (4.1%) and DLE (43%) than the control group, which suggested a compact π-π staking between the benzene ring of cinnamic alcohol and the PTX, along with the enhanced encapsulation efficiency.

**Table 1:** Drug loading content (DLC), drug loading efficiency (DLE) and particle size of CPLA-*blink*-PMPC and PLA-*blink*-PMPC micelles

Micelle	Particle Size (d.nm)		DLC (%)	DLE (%)
	Blank	PTX-loaded		
PLA-*Blink*-PMPC	140 ± 3	189 ± 4	3.8 ± 0.12*	40 ± 1.0**
CPLA-*Blink*-PMPC	135 ± 2	144 ± 3	4.1 ± 0.17*	43 ± 0.6**

* *P* < 0.01.

** *P* < 0.001. *n* = 6.

The morphology of CPLA-*Blink*-PMPC micelles was further investigated by TEM which was as shown in Fig. 4B. It can be seen that CPLA-*Blink*-PMPC micelles had an approximately spherical shape with a diameter of about 70 nm, which was smaller than that measured by DLS. The possible reason could be attribute to the shrinkage of the PMPC shell during sample preparation.

The responsive ability of PTX-loaded micelles was characterized by incubating the drug delivery platform under pH 5.5 and measuring the particle size of micelles with DLS at selected time intervals. As shown in Fig. 4C, the particle size distribution of micelles changed from unity to disorder with time went on, showing the structure of drug loaded micelles was gradually destroyed under acidic environment, which resulted in quickly release of the payload. The result in Fig. 4C was in accordance with the change of particle size in Fig. 4D. The micellar size increased from 74 to 225 nm after being incubated for 6 h at pH 5.5, suggesting the disassembly of pH-sensitive micelles, while the micelle size varied slightly at pH 7.4 for even 48 h (Supplementary Fig. S4). Therefore, the structure of these PTX-loaded micelles could be quickly disrupted under tumorous specific microenvironment, which would result in efficient drug release.

### *In vitro* release behavior of PTX from PTX-loaded micelles

**Figure 5:**
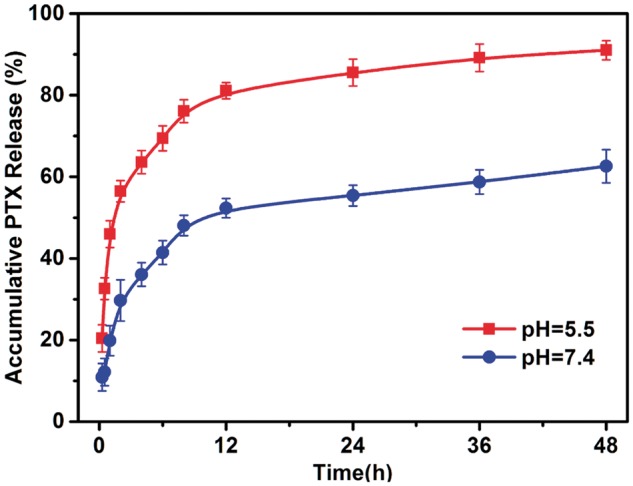
Accumulative release of PTX from drug loaded micelles at pH 7.4 and 5.5

The PTX-loaded micelles were expected to be stable during circulation *in vivo* while quickly release the drug under tumorous acidic condition due to the pH sensitive linkage. Therefore, the drug release behavior was carried out at pH 7.4 and 5.5 (Fig. 5). At pH 7.4, only 50% of PTX was released in 24 h and the initial burst release wasn’t clear, suggesting that the PTX-loaded micelles were stable at physiological pH. However, more than 90% of loaded PTX were released from micelles in 48 h when the drug loaded micelles were exposed to a pH of 5.5, which was ascribed to the breakage of benzoyl imine linkage, indicating that the PTX-loaded micelles could keep stable in systemic circulation but quickly release the drug and result in better antitumor effect.

### Cytotoxicity and cellular uptake

**Figure 6:**
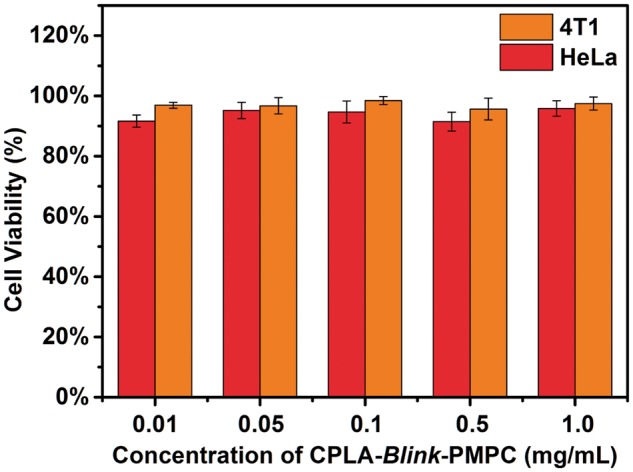
Cytotoxicity of HeLa and 4T1 cells incubated with various concentrations of blank CPLA-*blink*-PMPC micelles for 24 h

The *in vitro* cytotoxicity of blank CPLA-*Blink*-PMPC micelles and PTX-loaded CPLA-*Blink*-PMPC micelles were evaluated by CCK-8 assay against HeLa and 4T1 cells. As shown in Fig. 6, the blank CPLA-*Blink*-PMPC micelles showed negligible toxicity to both HeLa and 4T1 cells. The relative cell viability of blank micelles with different concentrations was all around 100% even if the concentration of CPLA-*Blink*-PMPC reached to 1 mg ml^−1^, which indicated the great biocompatibility of CPLA-*Blink*-PMPC. This great biocompatibility might owe to the biomimetic PMPC shells and the biocompatible PLA cores of micelles.

**Figure 7:**
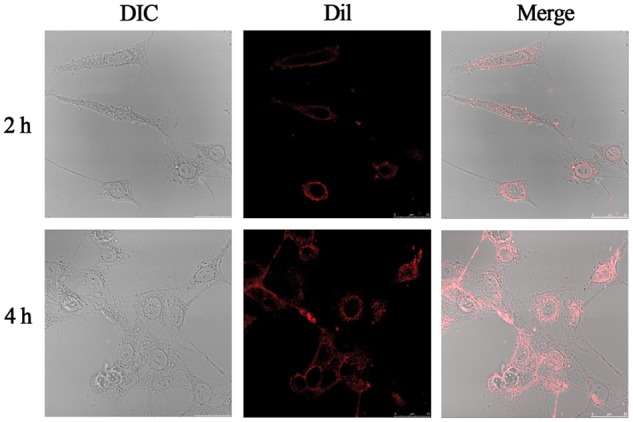
Confocal images of 4T1 cells incubated with dil-loaded micelles for 2 h and 4 h. From left to right: DIC, dil (red) and a merge of two images. The scale bars are 25 μm in all images

It was important for drug carriers to be efficiently internalized by tumor cells. In order to evaluate the cellular uptake of PTX-loaded micelles, fluorochrome Dil was loaded into the core of CPLA-*Blink*-PMPC micelles and the particle size of Dil-loaded micelles was measured by DLS to be 192 nm (Supplementary Fig. S3). Then the Dil-loaded micelles were incubated with 4T1 cells and observed with a confocal laser scanning microscopy (CLSM) at selected time intervals. According to Fig. 7, the red fluorescence of Dil was discovered in cytoplasm and the intensity of red fluorescence become stronger with the extension of the time, indicating that the CPLA-*Blink*-PMPC micelles could be effectively internalized by tumor cells and provide convenience for drug delivery in cytoplasm.

**Figure 8:**
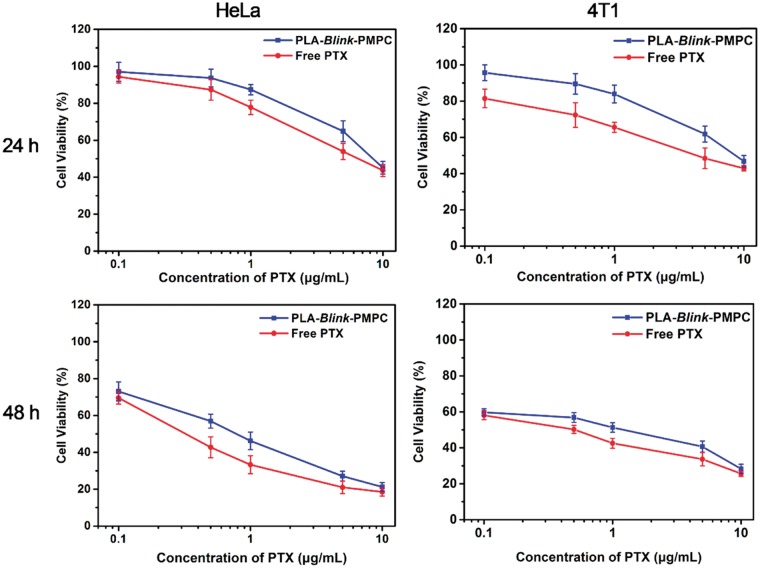
Cytotoxicity of HeLa and 4T1 cells incubated with various concentrations of PTX-loaded CPLA-*blink*-PMPC micelles and free PTX for 24 h and 48 h

To investigate the antitumor efficacy of PTX-loaded micelles, the cytotoxicity of PTX-loaded CPLA-*Blink*-PMPC micelles against 4T1 and HeLa cells was evaluated by CCK-8 assay. As shown in Fig. 8, the relative cell viabilities of the two cells displayed the concentration dependence. With the increasing concentration of PTX to 10 μg ml^−1^, the cell viabilities of both two cells decreased to 45% in 24 h and 25% in 48 h. Meanwhile, PTX-loaded micelles exhibited similar cytotoxicity as compared with free PTX in 24 h or 48 h, indicating the great anti-tumor efficacy of these PTX-loaded micelles owing to the efficient internalization and the pH-sensitivity. Therefore, these PTX-loaded CPLA-*Blink*-PMPC micelles would be a potential carrier for PTX delivery and cancer therapy.

## Conclusions

In this study, an amphiphilic CPLA-*Blink*-PMPC copolymer with an acid-labile switch was synthesized to develop pH sensitive polymeric micelles for PTX delivery. The PTX was loaded into the core of the micelles via hydrophobic interaction and the conjugation between PTX and cinnamenyl terminal group. These PTX-loaded micelles possessed a well-defined structure with narrow distribution, which was quite stable at normal physiological pH but quickly broke down in 6 h in tumor acidic environment and led to rapidly release of drug, resulting in improved antitumor efficacy. Moreover, the biomimetic micelles possessed effective cellular uptake and impressive antitumor efficacy as compared with free PTX. In conclusion, these polymeric micelles might be served as potential carriers for PTX delivery in cancer therapy.

## Supplementary Material

Supporting Information
