# Supplementary material for: A biomimetic and pH-sensitive polymeric micelle as carrier for paclitaxel delivery
Source: Regen Biomater. 2017 Aug 16;5(1):15–24. doi: 10.1093/rb/rbx023 (PMC5798030; doi:10.1093/rb/rbx023)
Supplement: Supporting Information [file supporting_information_1_rbx023.docx]

Supporting Information

**
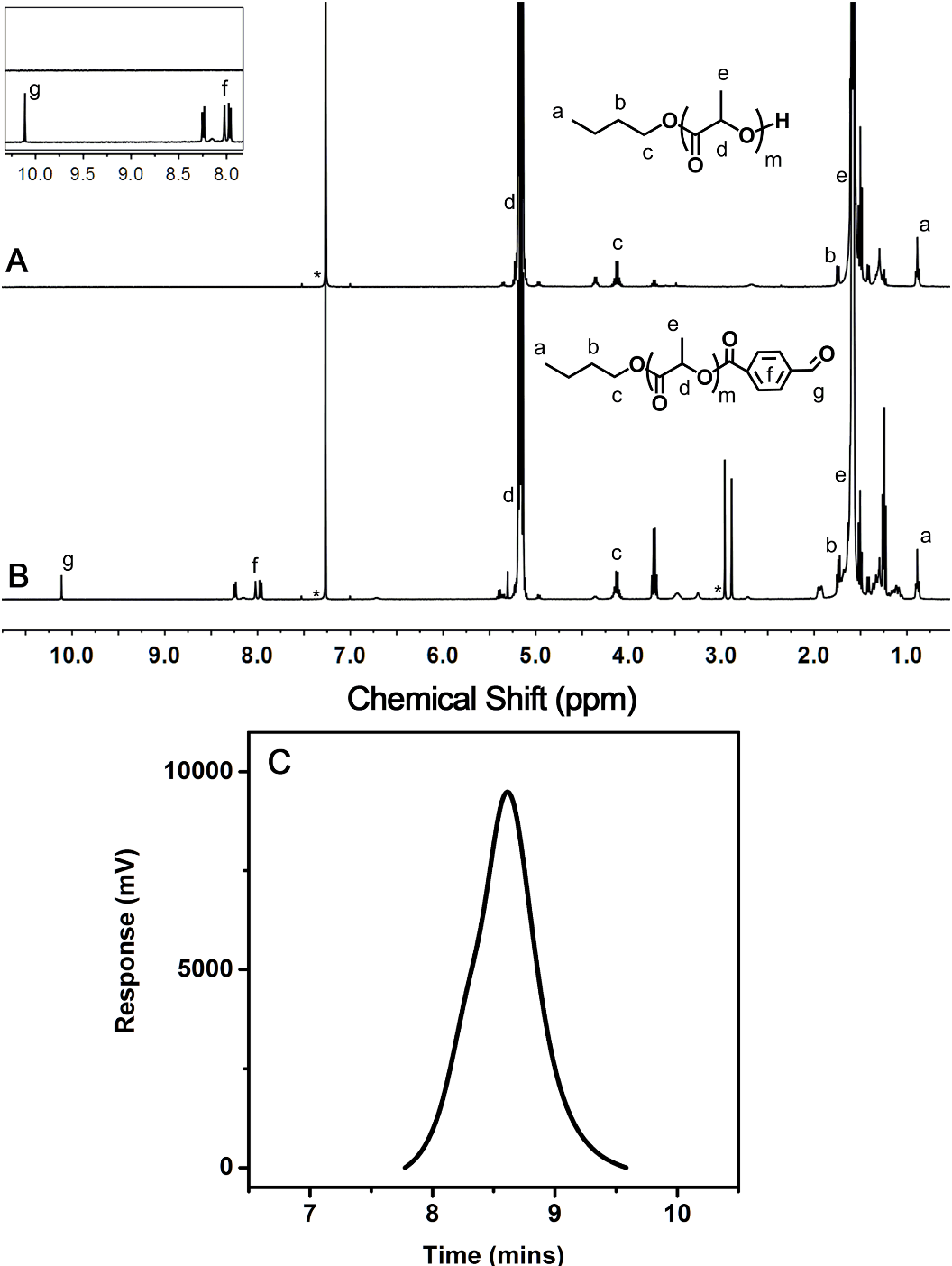
**

**Figure S1.** ^1^H NMR spectrum of PLA-OH (A), PLA-CHO (B) in CDCl_3_ and GPC spectra of PLA-CHO (C) in DMF.





**Figure S2.** ^1^H NMR spectrum of PLA- *Blink*-PMPC in CD_3_OD/DMSO-d_6_ (1/1, v/v).





**Figure S3.** Partical size of DiI-loaded micelles meatured by DLS.





**Figure S4.** Size variation of PTX-loaded micelles at pH 7.4 over time measured by DLS
